# The role of focused assessment sonography for trauma (FAST) on the outcomes in patients with blunt abdominal trauma following non-operative therapy: A cohort study

**DOI:** 10.1016/j.amsu.2022.104086

**Published:** 2022-06-25

**Authors:** Dewi Sukorini Wahyuningtias, Aditya Rifqi Fauzi, Eko Purnomo, Imam Sofi

**Affiliations:** Digestive Surgery Division, Department of Surgery, Faculty of Medicine, Public Health and Nursing, Universitas Gadjah Mada/Dr. Sardjito Hospital, Yogyakarta, 55281, Indonesia

**Keywords:** Blunt abdominal trauma, FAST, Non-operative therapy

## Abstract

**Background:**

The non-operative management of blunt abdominal trauma had a high success rate and is expected to reduce the length of hospitalization and patients' morbidity. Here, we aim to evaluate the outcomes of patients with blunt abdominal trauma after non-operative management and associate them with prognostic factors.

**Methods:**

We performed a retrospective analysis on patients with blunt abdominal trauma who received non-operative management (NOM) at our institution from April 2018 to April 2021.

**Results:**

Two hundred eleven patients were included in this study who underwent non-operative management. Most of the subjects (73%) were males, with male to female ratio of 2.7:1. Most patients aged 20–29 years old (29.4%), FAST negative (62.1%), minor injured (45%), successfully managed nonoperatively (98.6%), received no transfusion (38.9%), and injured due to traffic accident (80.1%). ISS was significantly associated with FAST (p = 0.028), while male gender, NLR, PLR, and blood transfusion did not (p > 0.05). The presence of external injury was associated with FAST results (p = 0.039), while the head, facial, thoracic, pelvic, and skeletal injuries did not (p > 0.05). We also found a significant correlation between blood transfusion and patient survival with NOM outcomes (p = 0.047 and p = 0.041, respectively). Furthermore, external injury significantly correlated with NOM outcomes (p = 0.042). Multivariate analysis showed that external and pelvic injury was significantly associated with NOM outcomes (p < 0.0001 and p = 0.036, respectively).

**Conclusions:**

The results of the FAST examination were not associated with the outcome of non-operative therapy. Moreover, the successful outcome of NOM might be affected by blood transfusions, the presence of external injuries, and pelvic injury.

## Highlights

• Non-operative management of blunt abdominal trauma had a high success rate.
• The results of the FAST examination will determine the patient's management.
• FAST examinations were not associated with the outcome of non-operative therapy.

## Abbreviations

CI: Confidence interval
POD: Postoperative day
FAST: focused assisted sonography for trauma
ISS: injury severity score
NOM: non-operative management
NOM-F: non-operative management-failure
NOM-S: non-operative management-success
SBP: systolic blood pressure
TRISS: trauma injury severity score
NLR: neutrophil-to-lymphocyte ratio
PLR: platelet-to-lymphocyte ratio
dHCT: delta hematocrit

## 1 Introduction

Trauma is the leading cause of world mortality, with 90% of the burden in the middle to low-income countries [1]. The abdominal injury occurred in 31% of patients with polytrauma. In Sleman district, Yogyakarta, Indonesia, in 2015–2016, data on traffic accidents with abdominal trauma alone reached 80% [2].

In one study, the non-operative management of blunt abdominal trauma had a high success rate (89.9%) and was also safe. In liver and spleen injuries in hemodynamically stable patients, conservative management can be performed regardless of the degree of damage [3]. With conservative management, the length of hospitalization is expected to be reduced and patient morbidity [3].

In abdominal trauma cases, a rapid and non-invasive examination is required, namely Focused Assessment Sonography for Trauma (FAST). The accuracy value was obtained at 99.4%, with a positive predictive value of 100% and a negative predictive value of 99.4%, so abdominal ultrasonography is considered an important and integrated part of managing major trauma patients. The results of the FAST examination in cases of abdominal trauma will determine the follow-up to the patient's management [4].

However, no studies delve deeper into the comparative information between FAST and the outcome of non-operative therapy in blunt abdominal trauma. This study aimed to evaluate the outcomes of patients with blunt abdominal trauma after non-operative management and associate them with prognostic factors, such as gender, age, laboratory parameters, radiology, and postoperative complications.

## 2 Methods

### 2.1 Patients

This retrospective study was conducted on patients with blunt abdominal trauma who received non-operative management (NOM) from April 2018 to April 2021. We included all patients with blunt abdominal trauma and performed FAST who received NOM at our institution. At the same time, the exclusion criteria were pregnant patients, patients who consumed routine anticoagulant medication, patients who refused treatment, patients who were hemodynamically unstable and underwent laparotomy, and incomplete medical records.

This study was approved by the Medical and Health Research Ethics Committee of the Faculty of Medicine, Public Health and Nursing, Universitas Gadjah Mada/Dr. Sardjito Hospital, Yogyakarta, Indonesia (#KE/FK/1162/EC/2021), and written informed consent were obtained from the patients. This study has been reported in line with the STROCSS criteria [5] and has been registered in the research repository of the Faculty of Medicine, Public Health and Nursing Universitas Gadjah Mada with Register ID: 202 205 118.

### 2.2 FAST assessment

Surgery residents performed the FAST in the emergency room. The procedure was conducted according to Advanced Trauma Life Support (ATLS). A positive TEST result is indicated by free intra-abdominal fluid, while the absence of free intra-abdominal fluid indicates a negative FAST result.

### 2.3 Non-operative management

Non-operative therapy is defined as managing patients during hospitalization with close supervision [3]—evaluation protocol by assessing vital signs, urine output, hemoglobin levels, and hematocrit. Repeat ultrasound examination or CT scan if there is a decrease in hemoglobin. Three bags of blood transfusion products must be given, abdominal distension, signs of infection, vomiting, haematuria, or tachypnoea. The patient is said to have successfully undergone non-operative therapy (NOM-S/non-operative management-success) if there are no signs of continued bleeding, infection, vomiting, haematuria, or tachypnoea. In other words, the patient is stable until the end of treatment and is considered to have failed if the patient management was converted to surgery (laparotomy) (NOM-F/non-operative management-failure).

### 2.4 Statistical analysis

We presented data as frequency (percentage) and mean. The association between variables was analyzed using Fisher-exact or chi-squared tests and *t*-test with 95% confidence interval (CI), followed by multivariate logistic regression analysis. The p-values < 0.05 were considered significant. The statistical analysis was performed using IBM Statistical Package for the Social Sciences (SPSS) version 21 (Chicago, USA).

## 3 Results

### 3.1 Baseline characteristics

We examined 242 medical records of patients recruited consecutively and excluded 31 subjects due to incomplete medical records. In total, we checked 211 subjects in the final analysis. Most of the subjects (73%) were males, with male to female ratio of 2.7:1. Most patients aged 20–29 years old (29.4%), FAST negative (62.1%), minor injured (45%), successfully managed nonoperatively (98.6%), received no transfusion (38.9%), and injured due to traffic accident (80.1%) (Table 1).

**Table 1:** Baseline characteristics of patients at our institution.

Characteristics	N	%
*Gender*		
Male	154	73%
Female	57	27%
*Age*		
≤20 years	12	5.7%
20–29 years	62	29.4%
30–39 years	38	18.0%
40–49 years	40	19.0%
50–59 years	27	12.8%
60–69 years	21	10.0%
70–79 years	10	4.7%
80–89 years	1	0.5%
*FAST*		
Positive	80	37.9%
Negative	131	62.1%
*ISS*		
Minor injury (ISS 1–9)	95	45.0%
Moderate injury (ISS 10–15)	62	29.4%
Severe injury (ISS 16–24)	50	23.7%
Extremely severe injury (ISS ≥25)	4	1.9%
*Intensive care requirement*		
Not required	117	55.5%
Required for 1–3 days	37	17.5%
Required for 4–7 days	24	11.4%
Required for >7 days	33	15.6%
*Conversion*		
Yes (NOM-F)	3	1.4%
No (NOM-S)	208	98.6%
*Blood transfusion*		
No	82	38.9%
≤2 PRC	54	25.6%
>2 PRC	75	35.5%
*Other injuries*		
Head injury	74	35.1%
Facial injury	41	19.4%
Thoracic injury	64	30.3%
Pelvic injury	43	20.4%
Skeletal injury	111	52.6%
External injury	74	35.1%
*Other surgery*		
Present	121	57.3%
Absent	90	42.7%
*Injury mechanism*		
Natural disaster	1	0.5%
Household injury	4	1.9%
Fall from height	32	15.2%
Traffic accident	169	80.1%
Criminality	5	2.4%
*Outcomes*		
Survived	185	87.7%
Dead	26	12.3%

*NOM-F: non-operative management failure; NOM-S: non-operative management success, PRC: packed red cell*.

### 3.2 Association between prognostic factors and FAST

Subsequently, we looked at the relationship between prognostic variables such as age, trauma onset, SBP, ISS, TRISS, NLR, PLR, dHCT, intensive care duration, length of stay, and FAST results. There was a significant association between trauma onset, SBP, ISS, and PLR with FAST results in blunt abdominal trauma patients following NOM (p < 0.05) (Table 2). We also found a significant association between the ISS, PLR, and blood transfusion with FAST results (p < 0.05) (Table 3). Multivariate analysis showed that ISS was significantly associated with FAST with an odds ratio (OR) of 2.086 (95% CI 1.081–4.028; p = 0.028), while male gender, NLR, PLR, and blood transfusion did not reach a significant level (Table 4).

**Table 2:** Univariate analysis between prognostic factors and FAST.

	FAST + (N = 80)	FAST – (N = 131)		
	Mean ± SD	Mean ± SD	*p*	95%CI
Age (year)	38.39 ± 16.01	40.48 ± 16.44	0.366	−6.64–2.46
Onset trauma (hour)	21.14 ± 38.75	11.12 ± 17.92	0.012*	2.26–17.76
SBP (mmHg)	119.09 ± 15.83	126.42 ± 24.85	0.019*	−13.45–−1.2
ISS	13.05 ± 7.48	10.43 ± 5.38	0.004*	0.87–5.37
TRISS	96.31 ± 10.05	97.27 ± 5.13	0.361	−3.02–1.10
NLR	11.87 ± 8.94	14.86 ± 12.87	0.069	−6.22–2.37
PLR	173.92 ± 102.49	230.73 ± 126.77	0.001*	−89.86–−23.75
dHCT	−3.86 ± 6.12	−3.96 ± 5.66	0.900	−1.531–1.739
Intensive care duration (day)	6.25 ± 6.22	8.61 ± 8.88	0.154	−5.62–0.89
Length of stay (day)	11.56 ± 7.28	12.54 ± 11.39	0.493	−3.78–1.83
Mortality	11.56 ± 7.24	10.76 ± 13.83	0.850	−7.743–9.325

*p-value considered significant if p < 0.05; SBP: systolic blood pressure; ISS: injury severity score; NLR: neutrophil to lymphocyte ratio; PLR: platelet to lymphocyte ratio; FAST: focussed assessment sonography for trauma; TRISS: trauma injury severity score; dHCT: delta hematocrit.

**Table 3:** Association and correlation between prognostic factors and FAST results.

	FAST				
	FAST + (N = 80)	FAST -(N = 131)	*r*	*p*	RR (95% CI)
*Gender* Male Female	54 (35.1%) 26 (45.5%)	100 (64.9%) 31 (54.4%)	0.096	0.161	0.769 (0.539–1.097)
*Age (year)* ≤30 >30	31 (39.2%) 49 (37.1%)	48 (60.8%) 83 (62.9%)	0.021	0.759	1.057 (0.743–1.505)
*ISS* ≤15 >15	52 (33.1%) 28 (51.9%)	105 (66.9%) 26 (48.1%)	0.166	0.014*	0.639 (0.455–0.897)
*NLR* ≤4 >4	11 (55%) 69 (36.1%)	9 (45%) 122 (63.9%)	0.113	0.098	1.522 (0.982–2.362)
*PLR* ≤137 >137	32 (48.5%) 48 (33.1%)	34 (51.5%) 97 (66.9%)	0.145	0.033*	1.465 (1.043–2.057)
*dHCT* ≤6 >6	55 (36.2%) 25 (42.4%)	97 (63.8%) 34 (57.6%)	0.057	0.406	0.854 (0.593–1.230)
*Intensive care* Not required Required	39 (33.6%) 41 943.2%)	77 (66.4%) 54 (56.8%)	0.097	0.155	0.779 (0.552–1.099)
*Blood transfusion* ≤2 units >2 units	44 (32.8%) 36 (46.8%)	90 (67.2%) 41 (53.2%)	0.137	0.045*	0.702 (0.500–0.987)
*Outcomes* Survived Dead	71 (38.4%) 9 (34.6%)	114 (61.6%) 17 (65.4%)	0.025	0.711	1.109 (0.634–1.939)

*p-value considered significant if p < 0.05.

**Table 4:** Multivariate analysis of the association between prognostic factors and FAST in patients with blunt abdominal trauma.

	p	OR (95%CI)
Male	0.243	1.468 (0.771–2.796)
ISS ≤15	0.028*	2.086 (1.081–4.028)
NLR ≤4	0.315	0.584 (0.204–1.668)
PLR ≤137	0.233	0.660 (0.333–1.307)
Blood transfusion ≤2 units	0.264	0.660 (0.767–2.643)

*p-value considered significant if p < 0.05.

### 3.3 Association between other injury mechanisms with FAST results

There was a significant association between external injury and FAST results (*p=*0.039) (Table 5). However, other injury mechanisms did not reach a significant level. Moreover, the external injury was significantly associated with FAST in multivariate analysis, with an OR of 2.097 (95% CI 0.614–2.022, p = 0.018). Thoracic injury almost reached a significant level in multivariate analysis with an OR of 1.839 (95% CI 0.979–3.454, p = 0.058), but not in univariate analysis (Table 6).

**Table 5:** Association between other injury mechanisms with FAST results.

	FAST				
	FAST + (N = 80)	FAST – (N = 131)	*r*	*p*	RR (95%CI)
*Head injury*			0.083	0.228	0.793 (0.540–1.167)
Present	24 (32.4%)	50 (67.6%)			
Absent	56 (40.9%)	81 (59.1%)			
*Facial injury*			0.112	0.103	0.661 (0.386–1.131)
Present	11 (26.8%)	30 (73.2%)			
Absent	69 (40.6%)	101 (59.4%)			
*Thoracic injury*			0.100	0.144	1.306 (0.922–1.851)
Present	29 (45.3%)	35 (54.7%)			
Absent	51 (34.7%)	96 (65.3%)			
Pelvic injury			0.065	0.342	1.217 (0.823–1.799)
Present	19 (44.2%)	24 (55.8%)			
Absent	61 (36.3%)	107 (63.7%)			
*Skeletal injury*			0.041	0.553	0.901 (0.638–1.272)
Present	40 (36%)	71 (64%)			
Absent	40 (40%)	60 (60%)			
*External injury*			0.141	0.039*	1.440 (0.614–2.022)
Present	35 (47.3%)	39 (52.7%)			
Absent	45 (32.8%)	92 (67.2%)			

*p-value considered significant if p < 0.05, RR, relative risk.

**Table 6:** Multivariate analysis of the association between injury mechanisms with FAST results.

	*p*	OR (95%CI)
Head injury	0.875	0.948 (0.485–1.852)
Facial injury	0.169	0.554 (0.23–1.286)
Thoracic injury	0.058	1.839 (0.979–3.454)
External injury	0.018*	2.097 (1.134–3.879)

*p-value considered significant if p < 0.05; OR, odds ratio.

### 3.4 Association between prognostic factors and outcomes of non-operative management

We did not find any significant difference between prognostic factors with outcomes of NOM (p > 0.05) (Table 7. However, we found a significant correlation between blood transfusion and patient survival with NOM outcomes (p = 0.047 and p = 0.041, respectively) (Table 8).

**Table 7:** Association between prognostic factors and outcomes of non-operative management.

	NOM-F (N = 3)	NOM-S (N = 208)	*p*	95% CI
	Mean ± SD	Mean ± SD		
Age (year)	28.67 ± 4.61	39.85 ± 16.33	0.238	−29.81–7.45
Trauma onset (hour)	4.67 ± 2.30	15.07 ± 28.23	0.525	−42.61–21.81
SBP (mmHg)	108.67 ± 28.02	123.86 ± 22.04	0.239	−40.53–10.15
ISS	13.33 ± 8.08	11.39 ± 6.36	0.602	−5.38–9.25
TRISS	98.71 ± 0.89	96.88 ± 7.43	0.671	−6.65–10.31
NLR	5.64 ± 3.68	13.85 ± 11.65	0.225	−21.50–5.09
PLR	172.05 ± 239.08	209.72 ± 119.59	0.594	−176.73–101.37
dHCT	−2.27 ± 7.0	−3.94 ± 5.82	0.622	−5.01-8.37
Intensive care length of stay (day)	1 ± 0	7.75 ± 7.93	0.622	−17.95–4.45
Length of hospitalization (day)	3.33 ± 3.21	12.31 ± 10.04	0.235	−20.42–2.49

*p-value considered significant if p < 0.05; SBP, systolic blood pressure; ISS, injury severity score; NLR, neutrophil-to-lymphocyte ratio; PLR, platelet-to-lymphocyte ratio; NOM-F, non-operative management failure; NOM-S, non-operative management success; TRISS, trauma injury severity score; dHCT, delta hematocrit.

**Table 8:** Association and correlation between prognostic factors with outcomes of NOM.

	Outcomes				
	NOM-F (N = 3)	NOM-S (N = 208)	r	*p*	RR (95%CI)
*Gender*			0.017	0.613	0.740 (0.068–8.008)
Male	2 (1.3%)	152 (98.7%)			
Female	1 (1.8%)	56 (98.2%)			
*Age (year)*			0.072	0.557	3.342 (0.308–36.260)
≤30	2 (2.5%)	77 (97.5%)			
>30	1 (0.8%)	131 (99.2%)			
*FAST*			0.011	0.679	0.816 (0.073–9.152)
Positive	1 (0.5%)	79 (37.4%)			
Negative	2 (0.9%)	129 (61.1%)			
*ISS*			0.112	0.162	0.172 (0.016–1.859)
ISS ≤15	1 (0.6%)	156 (99.4%)			
ISS >15	2 (3.7%)	52 (96.3%)			
*NLR*			0.097	0.259	4.775 (0.453–50.36)
≤4	1 (5%)	19 (95%)			
>4	2 (1%)	189 (99%)			
*PLR*			0.091	0.231	4.394 (0.406–47.60)
≤137	2 (3%)	64 (97%)			
>137	1 (0.7%)	144 (99.3%)			
*dHCT*			0.014	0.628	0.776 (0.072–8.40)
≤6	2 (1.3%)	150 (98.7%)			
>6	1 (1.7%)	58 (98.3%)			
*Intensive care requirement*			0.052	0.589	0.409 (0.038–4.447)
Not required	1 (0.9%)	115 (99.1%)			
Required	2 (2.1%)	93 (97.9%)			
*Blood transfusion*			0.156	0.047*	1.041 (0.995–1.088)
≤2 unit	0 (0%)	134 (100%)			
>2 unit	3 (3.9%)	74 (96.1%)			
*Outcomes*			0.195	0.041*	0.070 (0.007–0.748)
Survived	1 (0.5%)	184 (99.5%)			
Dead	2 (7.7%)	24 (92.3%)			

*p-value considered significant if p < 0.05.

### 3.5 Association between prognostic factors and NOM outcomes

Furthermore, we analyzed the association and correlation between other injury mechanisms and NOM outcomes in patients with blunt abdominal trauma. External injury showed a significant correlation with NOM outcomes (p = 0.042) (Table 9). Moreover, in multivariate analysis, external and pelvic injury showed significant association (p < 0.0001 and p = 0.036, respectively) (Table 10).

**Table 9:** Association between prognostic factors and NOM outcomes.

	NOM				
	NOM-F (N = 3)	NOM-S (N = 208)	r	P	RR (95%CI)
*Head injury*			0.88	0.222	1.022 (0.997–1.048)
Present	0 (0%)	74 (100%)			
Absent	3 (2.2%)	134 (97.8%)			
*Facial injury*			0.059	0.521	1.018 (0.998–1.039)
Present	0 (0%)	41 (100%)			
Absent	3 (1.8%)	167 (98.2%)			
*Thoracic injury*			0.079	0.336	1.021 (0.997–1.045)
Present	0 (0%)	64 (100%)			
Absent	3 (2%)	144 (98%)			
Pelvic injury			0.137	0.106	7.814 (0.725–84.174)
Present	2 (4.7%)	41 (95.3%)			
Absent	1 (0.6%)	167 (99.4%)			
*Skeletal injury*			0.046	0.604	0.450 (0.041–4.892)
Present	1 (0.9%)	110 (99.1%)			
Absent	2 (2%)	98 (98%)			
*External injury*			0161	0.042*	0.959 (0.916–1.005)
Present	3 (4.1%)	71 (95.9%)			
Absent	0 (0%)	137 (100%)			

*p-value considered significant if p < 0.05.

**Table 10:** Multivariate analysis between other injury mechanisms and NOM outcomes.

	p	OR (95%CI)
Head injury	0.997	10341888.81 (0)
Pelvic injury	0.036*	0.065 (0.005–0.833)
External injury	<0.0001*	∞

*p-value considered significant if p < 0.05.

## 4 Discussion

Here, we report the non-operative management outcomes of patients with blunt abdominal trauma following FAST examination. Our patient's demography showed 72% male and commonly young age. This is consistent with injury prevalence statistics issued by the Indonesian Ministry of Health in 2018, which revealed that the percentage of males (11%) and the age group 15–34 years (20.1%) were more prevalent [6].

The results of the univariate analysis on the characteristics of the research data obtained a mean age of 39.7 years; trauma onset of 14.9 h, which is consistent with previous studies that showed a mean age of 35 ± 17 years [7], but with a different trauma onset of 5.6 h [8]. This demonstrates that injuries often occur at productive ages. However, the commencement of trauma until medical care is administered is linked to access and availability of medical services in different trauma sites.

The ISS value in this research was 11.42 (95% CI 10.56–12.29) with an SBP value of 123.64 (95% CI 120.64–126.64), which differed from prior studies with an ISS mean of 17.742 and an SBP value of 99.66 [9]. This might be related to differences in the severity of the damage across patients.

There was a significant association between trauma onset, SBP, ISS, and PLR with FAST results in blunt abdominal trauma patients following NOM. This indicates that the longer the patient is brought to the trauma ercenter for treatment, the more likely intra-abdominal bleeding will develop, followed by a decrease in SBP, which indicates hemodynamic instability.

The results of this study are supported by a study that showed that the results of the study in hemodynamically unstable patients (SBP <90 mmHg) had a significantly positive FAST result (p < 0.05), which was later confirmed by CT-scan and found that 28% had a splenic injury [10]. Meanwhile, the onset of trauma or prolonged prehospital time will increase the mortality rate by 5.3%, 9.9%, and 19.4% for 30, 60, and ≥180 min of delay. For patients with bleeding, delay in treatment increased mortality by 1.6%, 1.8%, and 1.8% for delays of 30, 60, and 180 min, respectively. This is related to the need for resuscitation [11].

Notably, our results showed that high ISS scores were associated with FAST; this was supported by a study by Ma et al. [11] with a mean ISS score of 12 (range 1–75), and positive FAST was found in 33 of 270 patients (12%) with a sensitivity of 89% and specificity of 99%.

Our findings found a significant difference in the need for blood transfusions with FAST, which is similar to the findings of Yin et al. [12], who calculated the need for blood transfusions using the thromboelastography technique in patients with abdominal trauma, obtaining an average yield of 4 PRC bags (range 3–11.5; p < 0.001), and 4 FFP bags (range 2.9–9.8; p = 0.036). However, no particular studies have investigated blood needs in individuals with positive FAST. Because of intravascular to intraperitoneal transudation, the presence of substantial fluid resuscitation or transfusion will result in a false positive FAST appearance [13].

PLR is also significant in FAST findings, indicating that the PLR value tends to decrease in the positive FAST group compared to the negative FAST group. Although no studies have been conducted to investigate the association between PLR values and FAST outcomes in trauma, lower PLR values were identified in the non-survivor group compared to trauma survivors (51.3 vs. 124.2, p < 0.001), with PLR values ranging from 21.5 to 972.2 [14]. The PLR value defines the situation of platelets and lymphocytes, where platelets help maintain hemostasis by adhering to blood arteries and aggregating to create thrombi, limiting excessive bleeding. Platelets will produce inflammatory cytokines while interacting with neutrophils, T cells, and macrophages. Lymphocytes are the primary cellular components of the immune system, both cellular and humoral. Lymphocyte immune capability is negatively linked following trauma or bleeding, and a reduction in lymphocytes is connected with the development of sepsis in trauma patients [14].

Gender, age, TRISS value, NLR, dHCT, duration of stay in the critical care unit, and overall length of stay were not significantly different between groups with FAST findings. These reports were similar to a previous study by Rose [15], which suggests that the presence of free fluid (anechoic appearance) on FAST examination may include blood, ascites, urine, and normal intestinal fluids. The presence of free fluid (positive FAST), regardless of volume, has no bearing on the decision to operate as long as the patient is hemodynamically stable and capable of undergoing a CT scan. In the right lateral decubitus position, a small volume of 100 ml of fluid can be seen, and a minimum of 619 ml of free fluid in Morison's pouch can be detected. In contrast, depending on the examiner's ability, a minimum of 500 ml (mean 250–620 ml) is required for perihepatic and peri-splenic spaces. It is not necessarily about the patient's age and gender [15].

NLR levels were not linked with FAST outcomes in our investigation. Previous research has not confirmed this. However, in a study comparing NLR values in trauma situations, researchers discovered that an NLR increase was directly linked to a higher ISS value. Increased NLR >4 was more likely in severe trauma [16].

Then, the decrease in hematocrit value (dHCT) was not associated with FAST results, which contradicts the findings of Mossadegh et al. [16], who found that a decrease in hematocrit of 5% or more has a sensitivity of 35.87%, specificity of 69.45%, 20% positive predictive value, and 83.56% negative predictive value for abnormal FAST results. Another study found that a decrease in hematocrit of 6 points predicted bleeding in patients who had not received fluid resuscitation (sensitivity 89%; specificity 95%). A decrease in hematocrit of 4 points predicted bleeding with a sensitivity of 98% and a specificity of 71%. The research included 86% of patients who had suffered blunt trauma [17].

Regarding the NOM results, our study demonstrated that age, gender, ISS, and FAST results did not differ significantly in NOM success. This is supported by research by Raza et al. [3], which states that age, gender, comorbidities, and trauma mechanisms do not affect NOM outcomes.

In this study, the onset of trauma and SBP values were not significantly different from the success of NOM. This finding contradicts a previous Giannopoulos et al. [18] study, which found that SBP levels greater than 90 mmHg predict NOM treatment efficacy. According to the research, there was a significant difference in ISS scores between the groups who successfully received NOM therapy with a mean ISS of 16 (6–41) vs. the group that failed NOM with an ISS of 29 (14–29) p < 0.001. This discrepancy might be explained by the prior study's comparisons with individuals having surgical therapy. Another research indicated that for every 10 min of delay in trauma initiation, the likelihood of mortality rose by 9% and 4% once other confounding variables were included [11].

The importance of NLR, PLR, and changes in hematocrit (dHCT) to the effectiveness of NOM was insignificant. No research has examined the association between NLR and PLR values and the efficacy of conservative trauma therapy; however, one study found that NLR values on day 1 predicted overall survival over the first 30 days following trauma. The NLR threshold value reached is 4. Therefore a result greater than this level indicates higher mortality [16]. According to research conducted by Ke et al. [19] on PLR values in trauma patients admitted to the critical care unit, low PLR values were strongly related to mortality (non-survivors 124.3 ± 110.3 vs. survivors 150.6 ± 106.4; p < 0.001), followed by high lymphocyte values and low platelet counts. On the other hand, the NLR value did not alter much [19].

The study's findings revealed a significant relationship between the requirement for blood transfusion and the efficacy of NOM. According to Velmahos et al. [7], four independent risk indicators potentially indicate NOM therapy failure: non-liver (splenic or renal) damage, positive FAST findings, an amount of free abdominal fluid >300 ml as demonstrated by CT scan, and the necessity for blood transfusions when NOM fails. The absence of these risk variables predicts 98% success in 96% of patients. The reported NOM failure rate for solid organ damage was greater than prior retrospective investigations. Non-operative therapy with liver damage is less likely to fail than splenic or renal injury. If a blood transfusion is necessary, FAST is positive, and a large blood volume is seen on an abdominal CT scan, NOM should be managed under close supervision [7].

The length of stay (intensive room length of stay and total length of stay) was found to be insignificant to the success of NOM, which contradicts the findings of Ibrahim et al. [1], who found that patients undergoing NOM had a longer length of stay (8.29 ± 2.8 days; p = 0.012) than those undergoing operative management. In terms of ward selection, Giannopoulos et al. [18] research showed that blunt abdominal trauma patients with stable hemodynamic circumstances might be managed in a non-intensive room under careful surveillance. This is consistent with the findings of the present investigation. As seen by a CT scan, splenic damage with free fluid in multiple locations increases the likelihood of NOM failure [18].

Bivariate and multivariate analysis revealed that external injuries resulted in a significant difference in FAST outcomes, although head injuries, face injuries, thoracic injuries, pelvic injuries, and skeletal injuries did not. According to research, patients with high energy trauma (e.g., falling from a great height, being thrown from a car, traffic accidents, lower rib fractures) have ruptured dense visceral tissue and/or aortic rupture unless confirmed non-existent. A fracture of the lower ribs (abdominal ribs) increases the possibility of an intra-abdominal injury, and a FAST and a pelvic fracture should be done. As much as 40% of individuals with abdominal ribs have positive FAST results without any first indications on physical examination; hence FAST may be repeated 4 h if the prior test is negative [20].

Bivariate analysis revealed that external damage resulted in a significant difference in NOM outcome. Meanwhile, multivariate analysis revealed that the existence of pelvic and external injuries resulted in a substantial difference in NOM outcome. However, this is not the case with head injuries, face injuries, thoracic injuries, pelvic injuries, or bone injuries. The findings of this study are consistent with the findings of Bansod et al. [21]. They found that the existence of thoracic injuries, bone injuries, head injuries, and other exterior injuries did not affect the effectiveness of NOM.

Our study noted some limitations, such as mono-institutional, relatively small sample, and retrospective nature of the study. Thus, further prospective, multicentre studies with a larger sample size are necessary to clarify and confirm our findings.

## 5 Conclusions

The results of the FAST examination were not associated with the outcome of non-operative therapy. Moreover, the successful outcome of NOM might be affected by blood transfusions, the presence of external injuries, and pelvic injury. At the same time, the gender, age, ISS, NLR, PLR, dHCT, and intensive room requirements might not be associated with any outcomes.

## Provenance and peer review

Not commissioned, externally peer-reviewed.

## Ethical approval

This study was approved by the Medical and Health Research Ethics Committee of the Faculty of Medicine, Public Health and Nursing, Universitas Gadjah Mada/Dr. Sardjito Hospital, Yogyakarta, Indonesia (#KE/FK/1162/EC/2021).

## Please state any sources of funding for your research

The authors declare that this study had no funding source.

## Author contributions

Dewi Sukorini Wahyuningtias conceived the study. Aditya Rifqi Fauzi drafted the manuscript. Imam Sofii and Eko Purnomo critically revised the manuscript for important intellectual content. All authors read and approved the final draft. All authors facilitated all project-related tasks.

## Please state any conflicts of interest

No potential conflict of interest relevant to this article was reported.

## Registration of research studies

Research repository: Faculty of Medicine, Public Health and Nursing Universitas Gadjah Mada.

Register ID: 202 205 118.

## Guarantor

Imam Sofii.

## Consent

Written informed consent was obtained from the patients for publication of this research. A copy of the written consent is available for review by the Editor-in-Chief of this journal on request.

## Annals of medicine and surgery

The following information is required for submission. Please note that failure to respond to these questions/statements will mean your submission will be returned. If you have nothing to declare in any of these categories then this should be stated.
